# Intertarsal Joint Stabilization in a Bateleur Eagle* (Terathopius ecaudatus)* Using a Novel Application of a Braided Suture and Titanium Button System

**DOI:** 10.1155/2017/7373242

**Published:** 2017-12-10

**Authors:** Jenessa Gjeltema, Ryan S. De Voe, Larry J. Minter, Brian J. Trumpatori

**Affiliations:** ^1^Karen C. Drayer Wildlife Health Center and Department of Medicine and Epidemiology, University of California Davis School of Veterinary Medicine, One Shields Ave., Davis, CA 95616, USA; ^2^Environmental Medicine Consortium, North Carolina State University College of Veterinary Medicine, 1060 William Moore Dr., Raleigh, NC 27607, USA; ^3^Department of Animal Health, Disney's Animal Kingdom, 2901 Osceola Parkway, Lake Buena Vista, FL 32830, USA; ^4^Veterinary Division, North Carolina Zoo, 4401 Zoo Parkway, Asheboro, NC 27205, USA; ^5^Veterinary Specialty Hospital of the Carolinas, 6405 Tryon Rd., Suite 100, Cary, NC 27518, USA

## Abstract

A 32-year-old adult captive male bateleur eagle* (Terathopius ecaudatus)* with a history of laxity, degenerative joint disease, and varus deviation of the distal left hindlimb for several years was presented for evaluation of left hindlimb lameness and was diagnosed with chronic subluxation of the left intertarsal joint. After failing to improve with conservative management and pain medication, surgical stabilization of the joint was performed using a novel application of a braided suture and titanium button system. Unsatisfactory clinical improvement and postsurgical reevaluation indicated that the initial surgical stabilization was unsuccessful. The surgery was repeated, and the animal showed postsurgical improvement in intertarsal joint stability, weight-bearing, and lameness for a period of several years with use and adjustment of chronic pain medications. The novel surgical technique described in this case report represents an additional treatment option for management of avian intertarsal joint subluxations. Presurgical planning should consider the unique anatomic features and variability of the avian tarsometatarsus to avoid surgical complications.

## 1. Introduction

Luxation of the intertarsal joint in avian patients is often due to trauma or underlying developmental abnormalities such as avulsion of the attachment site for the flexor hallucis muscle, rupture or displacement of the tibial cartilage, displacement of the gastrocnemius tendon, or rupture of the collateral ligaments and joint retinaculum [1–3]. Joint instability may lead to progressive and potentially debilitating osteoarthritis due to the abnormal biomechanical forces applied to the joint, and correcting joint instability plays an important role in preventing or reducing the severity of osteoarthritic changes. Surgical management is currently recommended for intertarsal joint luxations in birds. Surgical options include direct repair or replacement of ligaments and tendons, external fixation to allow fibrosis of the periarticular tissues, application of stabilization implants, arthrodesis, and amputation [1–5]. Despite the current recommendations for surgical intervention, there is little information available describing or evaluating different surgical techniques for treating luxation of the avian intertarsal joint [3].

## 2. Case Presentation

An adult captive male bateleur eagle* (Terathopius ecaudatus)* estimated to be 32 years old and weighing 2.4 kg was presented for evaluation of left hindlimb lameness. The animal had been transferred from another institution 4 weeks prior to presentation with a history of osteoarthritis at the left intertarsal joint and an asymptomatic systolic heart murmur. Laxity and varus deviation of the distal left hindlimb at the intertarsal joint were first observed 2 years prior to presentation at the animal's previous institution. It had also been treated for several previous episodes of bilateral pododermatitis.

**Figure 1:**
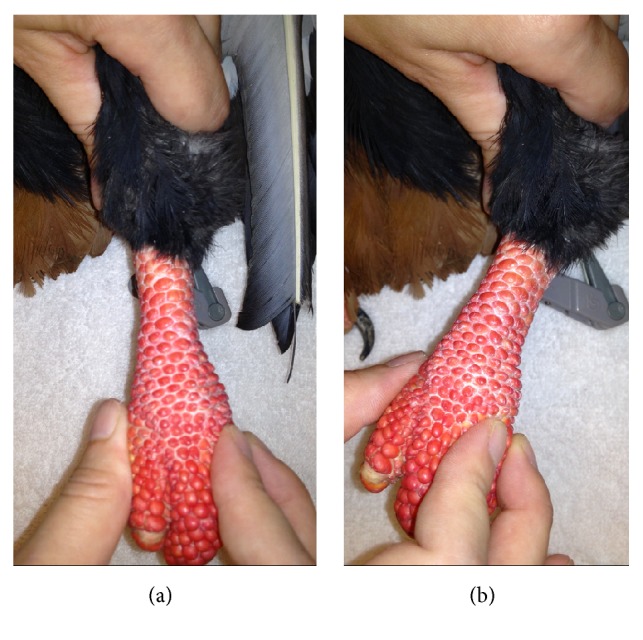
Subluxation of the left intertarsal joint with dynamic varus deviation of the distal limb in an adult male bateleur eagle* (Terathopius ecaudatus)*.

**Figure 2:**
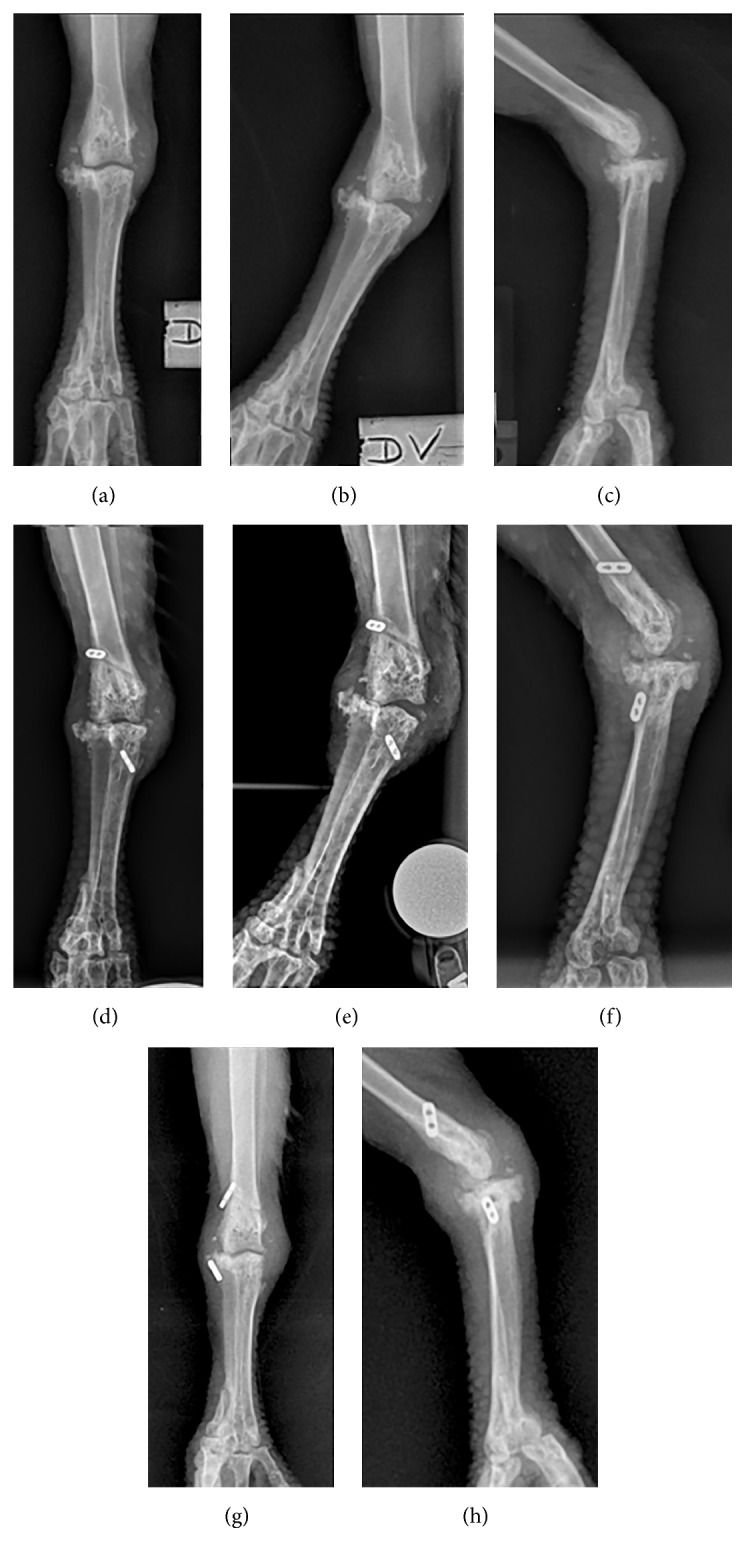
Sequential radiographs of an adult male bateleur eagle* (Terathopius ecaudatus)* with chronic left intertarsal joint instability performed prior to and following unsuccessful and successful surgical stabilization procedures using a novel application of a braided suture and titanium button system (Arthrex, Inc., Naples, USA). (a–c) Presurgical radiographs demonstrating subluxation of the left intertarsal joint, varus deviation of the distal limb, and associated degenerative joint disease at the left intertarsal joint using (a) standard dorsoplantar view, (b) stress dorsoplantar view (b), and standard lateral view. (d–f) Radiographs performed 7 weeks following unsuccessful surgical stabilization of the left intertarsal joint revealing suboptimal positioning of the stabilization implants and persistent subluxation of the left intertarsal joint using (d) standard dorsoplantar view, (e) stress dorsoplantar view, and (f) standard lateral view. (g and h) Postoperative radiographs following successful surgical stabilization of the left intertarsal joint showing resolution of subluxation and appropriate positioning of stabilization implants using (g) standard dorsoplantar view and (h) standard lateral view.

Several days after transfer into a new enclosure, the animal began to exhibit intermittent non-weight-bearing left hindlimb lameness and was prescribed tramadol (5.5 mg/kg bodyweight (BW) orally twice daily) for pain management. The animal failed to sufficiently respond to this treatment over the course of 1 month and began spending increased time in a sternal position or on the ground of the enclosure. Under general anesthesia with isoflurane gas and oxygen, a physical examination with radiographs, complete blood count, and plasma biochemistry was performed to evaluate the lameness. Physical examination revealed firm periarticular enlargement, reduced range of motion, subluxation, and dynamic varus deviation at the left intertarsal joint (Figure 1). No evidence of pododermatitis was observed at either foot. Orthogonal view radiographs of the pelvic limbs revealed muscle atrophy of the left hindlimb, moderate soft-tissue expansion around the left intertarsal joint with an uneven joint space that was widened at its lateral and narrowed at its medial aspects on the dorsoplantar radiographic view, and evidence of degenerative joint disease at this joint (Figure 2). A complete blood count and plasma biochemistry were considered unremarkable when compared to species reference values [6]. The chronic degenerative joint disease and instability likely associated with previous joint ligament or tendon rupture was thought to be the underlying cause of the lameness. The animal was prescribed meloxicam (0.5 mg/kg BW orally once daily) and continued on tramadol. A visual evaluation performed 2 weeks later revealed continued intermittent lameness of the left hindlimb with reduced weight-bearing and frequent placement of the limb in an abnormal extended position. Gabapentin (3 mg/kg BW orally once daily) was prescribed for additional pain management; however, progressive lameness of the left hindlimb persisted despite these conservative management efforts. Reevaluation was performed 10 weeks later when the animal was observed to be non-weight-bearing on the left hindlimb after several traumatic collisions within its enclosure. The patient was anesthetized as previously described. Physical examination findings were consistent with the animal's exam performed 10 weeks earlier, although bruising was present at the left ventral pelvic region, likely due to the recent observed collisions. Stress radiographs of the distal limbs were performed, confirming subluxation at the left intertarsal joint (Figure 2). Repeated complete blood count and plasma biochemistry revealed elevated creatine kinase (1175 U/L; Species 360 database reference values 133–795) consistent with muscle damage from soft-tissue trauma or capture and handling [6]. A support bandage was applied to the left intertarsal joint region, and the animal's prescriptions of tramadol, meloxicam, and gabapentin were continued. Based on physical examination findings, diagnostics, and the patient's failure to respond adequately to conservative management alone, surgical stabilization of the left intertarsal joint was scheduled for the following week.

**Figure 3:**
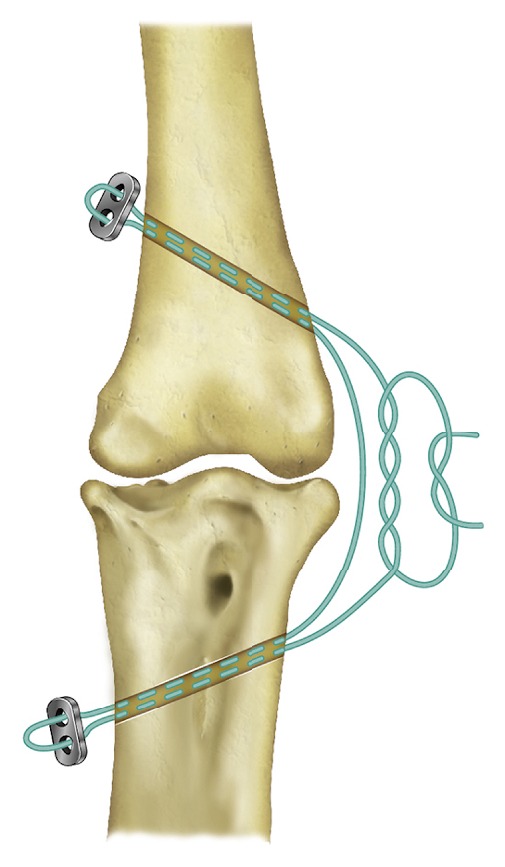
Illustration of the dorsal (anterior) aspect of the left intertarsal joint depicting novel application of a braided suture and titanium button system for surgical stabilization of left intertarsal joint instability in an adult male bateleur eagle* (Terathopius ecaudatus)*. The braided suture was passed from the lateral aspect of the intertarsal joint through two intraosseous tunnels created at the distal tibiotarsus and proximal tarsometatarsus. Each end of the suture was threaded through both holes of a 2-hole titanium button and passed back through each respective intraosseous tunnel. The suture was then tightened to an appropriate tension and secured using a surgeon's knot.

The animal was induced under general anesthesia as previously described, intubated, and maintained on isoflurane gas and oxygen throughout the surgical procedure. The left intertarsal joint was aseptically prepared, and sterile adhesive drape (Ioban™, 3M, St. Paul, USA) was applied to the limb. A 2 cm incision was made over the lateral aspect of the left intertarsal joint, and the soft-tissues were bluntly dissected from the distal tibiotarsus and proximal tarsometatarsus. A 30 ga. needle was placed into the intertarsal joint space to confirm its location, and a 2 mm drill bit was used to make an intraosseous tunnel through both cortices of the distal tibiotarsus extending proximomedially from the distolateral aspect of the metaphysis (Figure 3). A second intraosseous tunnel was produced at the proximal tarsometatarsus extending distomedially from the lateral aspect of the bone. Incisions were made over the medial aspects of the tibiotarsus and tarsometatarsus at both exit points of the intraosseous tunnels, and soft-tissues were bluntly dissected away from the underlying bone to allow application of the stabilization implants. A flexible suture passer was used to shuttle a single strand of braided suture material (#2 Arthrex FiberWire®, Arthrex, Inc., Naples, USA) from the lateral entry points of both intraosseous tunnels to the medial exit points at both bones (Figure 3). Each end of the suture material was threaded through both holes of a 2-hole titanium suture button (Arthrex, Inc., Naples, FL, USA) and back through its respective intraosseous tunnel to exit at the lateral aspect of the intertarsal joint. The buttons were positioned against the bones while the braided suture material was tied with the joint in a neutral position. Range of motion and varus/valgus stability of the intertarsal joint were assessed prior to tying of the suture to ensure appropriate joint stability and range of motion. Closure of the soft-tissues and skin was performed using 3-0 polydioxanone suture in a simple interrupted pattern. Postoperative radiographs revealed that the titanium button at the tarsometatarsus was positioned craniolaterally to what was considered ideal; however, the joint appeared stable during manipulation. The animal received perioperative butorphanol (2 mg/kg BW intramuscularly), meloxicam (0.5 mg/kg BW intramuscularly), clindamycin (20 mg/kg BW intravenously), enrofloxacin (15 mg/kg BW subcutaneously diluted 1 : 10 in lactated ringer's solution), and intraoperative lactated ringer's solution (10 ml/kg/hr BW intravenously). The patient continued tramadol, meloxicam, and gabapentin and was also prescribed prophylactic clindamycin (20 mg/kg BW orally once daily) and enrofloxacin (20 mg/kg BW orally once daily) for 7 days.

Initial observations made during the first 3 weeks of postoperative recovery revealed gradual initial improvement in weight-bearing with significant persistent lameness. At 5 weeks after surgery, the animal was observed spending the majority of its time in sternal recumbency with reluctance to stand, and a reevaluation with the consulting veterinary surgical specialist was scheduled for the following week. The animal was induced, intubated, and maintained under general anesthesia as previously described. Radiographs confirmed suboptimal positioning of the previously placed joint stabilization implants, and persistent subluxation of the left intertarsal joint was demonstrated in stress radiographic views (Figure 2). The previous surgical stabilization was considered unsuccessful, and a second joint stabilization surgery was elected. The patient was aseptically prepared, consistent with the initial surgical procedure. Incisions were made at the medial and lateral aspects of the left intertarsal joint, and evaluation of the previously placed stabilization implants revealed that the distal titanium suture button had become unsecured from the intraosseous tunnel at the medial tarsometatarsus, leading to surgical stabilization failure and subsequent postoperative laxity at the joint. The previously placed implants were removed, and the surgical stabilization procedure was repeated similarly to what was described for the first surgical procedure. The intraosseous tunnel at the distal tibiotarsus was re-used, and the intraosseous tunnel at the proximal tarsometatarsus was evaluated, was determined to have widened, and was revised. A 1.143 mm diameter K-wire was passed from proximolateral to distomedial across the proximal tarsometatarsus. Suture material (#5 Arthrex FiberWire, Arthrex, Inc., Naples, USA) was threaded through the intraosseous bone tunnels and titanium buttons as described for the first surgery. The suture was tightened with the aid of a suture tensioner (Arthrex, Inc., Naples, FL., USA) to 5 kg, and the joint was cycled to ensure stability and range of motion. The tensioner was then removed and the suture was tied. A two-layer closure of the soft-tissues and skin was performed using 4-0 poliglecaprone suture in cruciate and simple continuous patterns. Lidocaine (1 mg/kg BW) was administered as an incisional block, postoperatively. The animal received perioperative butorphanol (0.5 mg/kg BW intramuscularly), meloxicam (0.5 mg/kg BW intramuscularly), and enrofloxacin (10 mg/kg BW subcutaneously) administered in lactated ringer's solution (40 ml/kg BW subcutaneously). Postoperative examination revealed only mild medial subluxation of the left intertarsal joint during flexion that was comparable to that observed at the contralateral limb. No significant medial subluxation was elicited with the joint in extension. Postoperative radiographs indicated appropriate positioning of the stabilization implants (Figure 2). Prophylactic enrofloxacin (15 mg/kg BW orally once daily) and clindamycin (20 mg/kg BW orally once daily) were prescribed for 28 days. Meloxicam, tramadol, and gabapentin were continued as previously prescribed, and activity restriction with lowered perches was implemented for 6 weeks.

Postsurgical visual and physical evaluations were performed periodically over the next 6 months. At 3 weeks after the second surgery, the bird was noticed spending less time in sternal recumbency than following the initial surgery, although the animal continued to have noticeable lameness at the left hindlimb. A postsurgical reevaluation performed under general anesthesia at 7 weeks revealed improved medial-lateral stability at the left intertarsal joint, although the animal continued to exhibit lameness at the left hindlimb. At 14 weeks following the second surgery, the animal was observed perching normally with good weight-bearing on both hindlimbs. Only slight intermittent favouring of the left hindlimb was observed during ambulation. Due to clinical improvement, the animal was weaned from gabapentin at 20 weeks without any increase in lameness or time spent in sternal recumbency but was maintained on tramadol and meloxicam for management of chronic pain related to the animal's underlying degenerative joint disease. At 6 months following the second surgery, the animal was using the limb well during perching and ambulation with only mild occasional favouring of the limb observed.

Periodic examinations, observations, and reports from animal caretakers indicated overall improvement in the animal's mobility and use of the limb for several years following the second surgical stabilization and ongoing treatment with pain medications. Only mild occasional lameness of the left hindlimb was observed during this time. Three years after the second surgical stabilization was performed, the animal developed a more pronounced lameness. Evaluation of the animal at this time revealed radiographic progression of degenerative joint disease at the left intertarsal joint, although no change in joint stability was noted. A complete blood count and plasma biochemistry were considered unremarkable when compared to species reference values [6]. The animal's medications were adjusted with meloxicam administered at 1 mg/kg BW orally twice daily and tramadol at 10 mg/kg BW orally twice daily. The animal responded positively to this change and was maintained on these medications to better manage the chronic pain associated with the condition.

## 3. Discussion

This case report describes the use of a braided suture material and titanium button implant system to achieve joint stabilization in a bateleur eagle with chronic intertarsal joint instability and associated lameness. Subluxation of the left intertarsal joint observed in the present case was presumed due to trauma, although a specific traumatic event was never observed. Varus displacement of the distal limb at the level of the intertarsal joint without significant rotational displacement is suggestive of disruption of the lateral collateral ligament and joint retinaculum. Due to the chronicity of the condition with periarticular fibrosis, it is unclear if and to what degree additional ligaments or tendons were involved.

Due to the patient's large size, chronicity of the instability, and the suspected underlying etiology of trauma, surgical stabilization using implants was elected. One technique has been previously described for the intertarsal joint in which application of a polyester fiber suture through mediolaterally oriented intraosseous tunnels at the tibiotarsus and tarsometatarsus was performed followed by tying of the ends of the suture together so that the implant encircled the joint along the frontal plane [1]. Although this technique corrected underlying instability, the biomechanical forces created by this repair technique could lead to abnormal compressive force applied to the joint.

In order to more accurately restore the function of the injured tendons and ligaments while also reducing bending and frictional forces that may contribute to implant failure over time, an alternative surgical technique using novel application of a braided suture anchor and titanium buttons was utilized for this case. The orientation and positioning of the stabilization implant materials allows for distribution of tension arising from the tightened suture to the titanium button implants located at the medial aspect of the joint rather than encircling and compressing the joint. Additionally, the FiberWire suture used in this case is made up of a core of ultrahigh molecular weight polyethylene material with a braided outer layer of combined polyester and polyethylene fibers that has demonstrated greater strength, knot security, and resistance to bending abrasion failure than many other suture materials, including polyester [7, 8]. The technique performed in this case is similar to that reported for correction of stifle, hip, and shoulder joint instability in the dog [9–11] as well as for a variety of applications in humans.

The initial surgery to correct the intertarsal joint instability in this patient was unsuccessful. The tarsometatarsus bone is an anteriorly posteriorly compressed shape with a longitudinally oriented concave caudal aspect that accommodates the flexor tendons in some birds. This appears to be a prominent anatomical feature in many raptor species that can complicate the surgical approach at this bone [2, 12]. The concave shape of the bone in this case may have contributed to suboptimal intraosseous tunnel positioning and persistent joint instability in this patient, although the joint initially appeared stable during manipulation at the time of the first surgery. It is also possible that postsurgical activity by the animal may have contributed to implant failure, especially if the intraosseous bone tunnel was structurally weak due to suboptimal positioning. Several attempts were required to achieve an appropriately positioned intraosseous bone tunnel in the tarsometatarsus during the animal's second surgical procedure, and postsurgical radiographs demonstrated the improved and more appropriate position of the titanium buttons at the medial aspect of the joint following the second surgery. This highlights the importance of considering the specific anatomic features of the tarsometatarsus during intertarsal joint stabilization surgery to avoid complications including suboptimal intraosseous tunnel positioning or trapping of flexor tendons by stabilization implant materials [2, 12]. While not used in the current case, computed tomography may be a helpful diagnostic tool that could provide additional information about joint structures and could aid in development of the surgical approach in bird species with varied tarsometatarsal anatomy.

One goal of correcting joint instability is to decrease abnormal biomechanical forces applied to the joint to prevent or reduce the severity of osteoarthritic changes over time. The animal of the current case had radiographic evidence of degenerative joint disease affecting the left intertarsal joint prior to surgical stabilization that was likely due to chronic joint subluxation. The surgical intervention was successful in mitigating the animal's presenting clinical signs for several years following surgery. However, despite achieving apparent increased joint stability using this surgical technique, reevaluation of the joint indicated progression of degenerative joint disease 3 years after the surgery was performed. It is unclear whether surgery was successful in reducing overall progression of degenerative changes or precipitated the progression of the osteoarthritis in this case. It is also unclear whether earlier intervention and stabilization of the joint would have yielded more favourable long-term results.

In addition to surgical correction of the intertarsal joint instability, the animal of this case was prescribed meloxicam, tramadol, and gabapentin for chronic pain management over the course of several years. Some avian species, such as vultures within the* Gyps* genus, have had significant adverse reactions to some nonsteroidal anti-inflammatory medications. While the long-term effects and tolerance of the medications prescribed in the current case have not been fully elucidated, studies have demonstrated safety of meloxicam administration in several avian species, including those sensitive to other nonsteroidal anti-inflammatory medications, over shorter administration durations [13–16]. No significant adverse clinical signs, clinicopathologic derangements, or radiographic abnormalities attributed to the administration of these medications were noted for the animal in this case.

The novel surgical technique described in this case report represents one option for stabilization of the intertarsal joint that may provide certain advantages over other surgical and management methods in select cases of avian intertarsal joint luxation. Presurgical planning should consider the potential challenges posed by anatomic variability of the tarsometatarsus to avoid surgical complications. Directed studies evaluating the biomechanical forces applied by and clinical outcomes of different joint stabilization techniques could be helpful in guiding clinical decisions related to intertarsal joint stabilization in the future.
